# Karl Wilhelm Kupffer And His Contributions To Modern Hepatology

**DOI:** 10.1186/1476-5926-2-S1-S2

**Published:** 2004-01-14

**Authors:** Kenjiro Wake

**Affiliations:** 1 Professor Emeritus, Tokyo Medical and Dental University, Liver Research Unit, Minophagen pharmaceutical Co., Ltd., 3-2-7, Yotsuya, Shinjuku-ku, Tokyo 160-0004, Japan

## Opening Lecture

It is a great honor for me to give the opening lecture at the 11^th ^International Symposium on Cells of Hepatic Sinusoid and Their Relation to Other Cells to commemorate the centennial of Karl Wilhelm Kupffer's death. I am happy to offer my thanks to Prof. Robert McCuskey, and members of the Organizing Committee of the Symposium for inviting me to this memorable event in the long history of liver studies.

**Figure 1:**
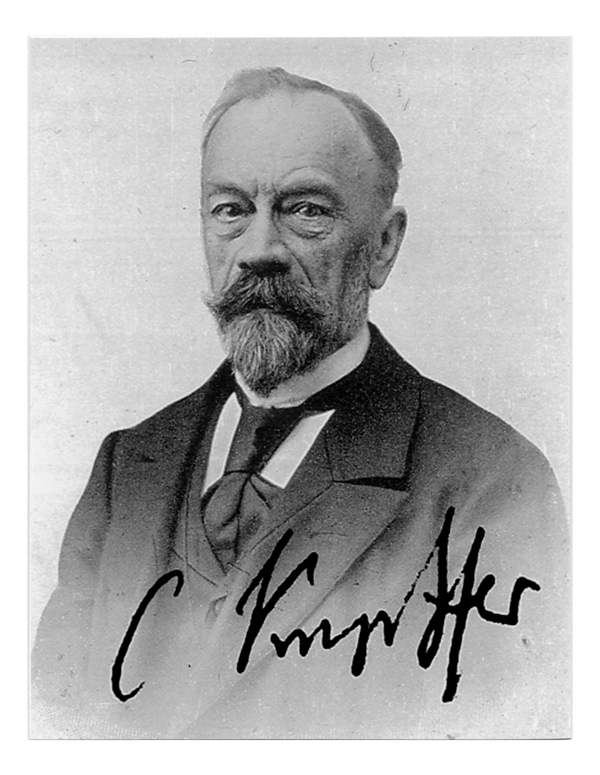
Portrait of Kupffer.

Karl Kupffer (Fig. 1) opened the major door to the fantastic, but complex universe of the hepatic sinusoid. During a century after his death, scientific approaches on the sinusoidal cells in the liver have undergone pronounced ups, twists, downs, and ups. Particularly stunning progress has been made during last three decades. The International Symposium on Cells of Hepatic Sinusoid, since the 1^st ^meeting held at Noordwijkerhout, The Netherlands in 1977, has produced many new ideas and perspectives on these interesting subjects of the liver.

Celebrating the centennial of Kupffer's death is a timely occasion to reassess how close to and at the same time, how far his observations and conceptions are from our present knowledge.

First, I would like to mention Kupffer's professional career briefly. And then, I shall discuss Kupffer's scientific contributions to our state-of the art views of the sinusoidal cells in the liver.

Karl Wilhelm Kupffer was born on November 14, 1829, at Lesten, near Mitau in Kurland; now belong to Latvia [1]. Karl was the first son of a pastor Karl Hermann Kupffer. Kupffer's family lived in a rectory with a wide garden leading into a meadow surrounded with a lake and large forest. The neighboring nature provided a good playground for children.

His parents didn't restrict freedom of their children, but taught them discipline. Karl learned reading and writing from his mother, and mastered all subjects of the gymnasium-course with the help of his father. He also took private lessons in French twice a week.

Nineteen-year-old Kupffer passed the qualifying examination to be a university student and entered Medical School of Dorpatt (at present Tartu) in 1848. After graduation, he went back to Kurland and started to work as a home-doctor. But he was not satisfied with the job and returned to Dorpatt Medical School to be an assistant of the Department of Anatomy. Under Prof. Bidder's guidance, he investigated structures of the central nervous system; the title of his dissertation [2] was "Spinal cord of a frog, with special reference to the gray matter."

This study was followed by his research on the development of the spinal cord. He described that dorsal-root nerve fibers entered the spinal cord and contributed to the formation of the marginal layer of the white matter, and that the ventral root fibers were derived from the nerve cells in the ventral horn.

After doing what we now call post-graduate study, Kupffer became Associate Professor at Dorpatt. Then, he was appointed to the Chair of Anatomy in the University of Kiel in 1866, and there he discovered the 'Sternzellen', hepatic stellate cells, using the gold chloride method. After publicizing this discovery, he moved to the University of K–nigsberg. In 1880, he was invited to the University of Munich to work as the Chair of Anatomy until he retired in 1901.

Through his visits in Wien, Berlin and G–ttingen in 1856-1857, to study physiology, he was greatly influenced by two famous physiologists, Dibois-Reymond and Johaness M–ller.

Karl Kupffer was fundamentally a neuroanatomist or embryologist. He may be called a pioneer of comparative embryology. Topics of his search were the fertilization, early differentiation of mesoderm, development of the brain, kidney, spleen and pancreas. In addition, he was interested in the innervation of exocrine glands, such as liver and pancreas.

When he was in K–nigsberg, he got a chance to measure the cranium of Immanuel Kant, a great philosopher, when his grave was dug up for reinvestigation. That was the only anthropological study for Kupffer [3].

During his long scientific career, he published over 60 articles, including review papers and books. However, the original papers concerning 'Sternzellen' in the liver were only two [4,5]. His investigation on the liver cells was just one incident in his eventful lifeworks.

As a matter of course, the most important contribution of Kupffer to us was the discovery of the stellate cells in the liver. He attempted to demonstrate nerve fibers in the liver, using the Gerlach's gold chloride method. His efforts bore no fruit. However, he found the star-shaped cells by chance. They were stained black with gold chloride, distributing in the liver lobule. He named these cells 'Sternzellen (stellate cells)', and reported them in the form of a letter to Prof. Waldeyer in 1876 [4].

He thought that the stellate cells were located perisinusoidally, and probably belonged to the Waldeyer's perivascular connective tissue cells or adventitia cells.

When Kupffer moved to Munich University, a doctor-course student, Paul Rothe joined his laboratory. Rothe used the same gold chloride method and confirmed the existence of stellate cells not only in the liver of mammals but also in the chick liver. Rothe's study [6] was published as a dissertation of Munich University in 1882, with a dedication to Prof. Kupffer.

Kupffer read another paper [7] on the 'so-called stellate cells' at the 12^th ^Anatomical Congress held in Kiel, April 17-19, 1898. This paper was published at full length in the next year [5]. He injected India ink or sheep erythrocytes to rabbits intravenously, and compared the India ink- or erythrocyte-uptake cells with the stellate cells in gold chloride preparations. On the basis of his observation and consideration, he changed his earlier opinion, and concluded that the stellate cells were the special endothelial cells of the sinusoids, which incorporate foreign substances.

As I pointed out in 1971 [8], Kupffer's new concept contained a serious misunderstanding. However, his new concept was accepted widely and influenced to the development of the theory of reticulo-endothelial system (RES). Vital staining, using lithium carmine, was extensively examined by Kenji Kiyono (1914) [9]. According to Kiyono, lithium-carmine-uptake cells consisted of two categories, 1) histiocytes that are free round or amoeboid cells, and 2) cells of the reticulo-endothelium. He thought that both cells had a common origin. Taking Kiyono's concept and the theory of the "endothelialer Stoffwechselapparat" [10] into consideration, Ludwig Aschoff (1924) proposed a new cell system in the body, called it the 'reticulo-endothelial system' [11].

In 1928, Zimmermann reported three kinds of cells in and around the sinusoids of the liver; endothelial cells, endocytes, and pericytes [12]. In 1898, at the Academy of Kracow in Poland, Browicz reported pear-shaped cells hanging in the lumen of the sinusoid [13]. Browicz's cells and Zimmermann's endocytes appear to be the same as the cells which we now called Kupffer cells. RES devotees believed that the cells hanging in the sinusoidal lumen were derived from the activated endothelial cells via the transitional cells [14-16].

Clear-cut distinction between sinusoidal endothelial cells and Kupffer cells in electron microscopy was brought by Eddie Wisse [17] in Leiden in 1970. Based on data of the bone marrow chimeras, parabiosis, and autoradiography, Ralph van Furth in Leiden proposed a new concept, the 'mononuclear phagocyte system' (MPS) [18]. In his comprehensive review on the MPS theory, the macrophage population is renewed by the influx of circulating monocytes. Since then, the RES has been replaced by the MPS.

On the other hand, since the beginning of the last century the presence of the Kupffer's original stellate cells had been deleted from textbooks, until I re-discovered them in 1971 [8]. A careful re-examination of Kupffer's gold chloride method and a concomitant application with silver impregnation methods, fluorescence, and electron microscopy revealed that the Kupffer's original stellate cells were not the sinusoidal endothelial cells nor phagocytic Kupffer cells, but they are perisinusoidal cells storing vitamin A, and they are identical to Zimmermann's 'pericytes' (1923) [19], 'Ito's 'fat-storing cells' (1951) [20], and Suzuki's 'interstitial cells' (1958) [21] (See Wake, 1980 [22]).

In the following, I would like to compare and contrast old findings and interpretations by Kupffer and Rothe, with the modern concepts.

(1) 'Gold staining of the stellate cells' (Kupffer 1876) [4]

The gold staining allows selective staining of vitamin A-storing stellate cells in the liver and extrahepatic organs. The sensitivity of the reaction is comparable to that of the fluorescence method for vitamin A. Thus, gold chloride reaction may be used as a histochemical method to detect vitamin A-storing lipid droplets. Using improved Kupffer's gold method, high quality gold preparations can easily be obtained (Wake et al. 1986) [23].

(2) 'Clear inclusion-bodies in the cytoplasm of the stellate cells' (Rothe 1882 [6]; Kupffer 1899 [5])

Rothe pointed out clear round inclusion bodies in the black-stained cytoplasm of the stellate cells. He thought these bodies to be 'small nuclei' of the stellate cells. Kupffer, in the second paper published in 1899, supposed that clear bodies might be the negative image of 'phagocytosed erythrocytes' due to his confusion of the stellate cells with the phagocytic cells.

I proved that the 'inclusion bodies' of the stellate cells were lipid droplets containing vitamin A (Wake, 1971) [8]. Gold precipitations occur primarily on the surface of these lipid droplets. Two types of lipid droplets are seen. Type I lipid droplets are membrane bound, and variable size, but always smaller than type II droplets, which have no membrane (Wake, 1974) [24]. The hepatic stellate cells of a pig and a hamster have only one or two large lipid droplets, while those cells of a rat, mouse, rabbit, and human have several smaller lipid droplets. The presence of fat-droplets were reported by Ito (1951) [20] in his 'fat-storing cells'. He thought that the 'fat' might be derived from glycogen in the cytoplasm.

Before the rediscovery of the original stellate cells, researchers believed that vitamin A was stored in the phagocytic Kupffer cells [25,26]. Because under a fluorescence microscope, vitamin A fluorescence is released from disseminated cells in the hepatic lobules, so they thought that disseminating cells were no different from the Kupffer cells.

Rune Blomhoff and his group (1982) in Oslo opened the door of intrahepatic metabolic pathways of vitamin A [27]. This investigation could not be realized without the development of the cell separation method of each sinusoidal cell. Dick L. Knook and his group (1984) in Leiden developed the cell separation method of stellate cells [28]. This method has accelerated in vitro studies to explore the biochemical and molecular analysis of these cells.

(3) 'Cytoplasmic processes of the stellate cells and closed relations of the stellate cells with endothelial cells and also with parenchymal cells' (Kupffer, 1876) [4].

Kupffer and Rothe described clearly that the stellate cells were located perisinuoidally and always attached to the sinusoidal wall and also to the parenchymal cells with cytoplasmic processes.

The cytoplasmic processes of the stellate cells can be demonstrated more prominently by the Golgi's silver impregnation method (Zimmermann, 1928 [19]; Wake, 1988 [29]), Bielschowsky's silver impregnation method (Suzuki, 1958) [21], immunohistochemical methods for desmin (Yokoi et al. 1984) [30], alpha-smooth muscle actin (Ramadori et al., 1990) [31] and GFAP (Niki et al., 1996) [32], and SEM after NaOH maceration method (Takahashi-Iwanaga and Fujita, 1986 [33]; Wake, 1988 [29]).

Golgi's silver impregnation method is the best method for demonstrating of whole view of long cytoplasmic processes of the stellate cells. The subendothelial processes encompass the endothelial cells of the sinusoid. These profiles suggest that the stellate cells are contractile in nature to regulate the sinusoidal blood flow and to accelerate the fluid exchange between the sinusoidal lumen and the space of Disse via endothelial fenestration (Wake, 1995) [34]. In 1992, Norifumi Kawada, we stayed at Karl Decker's laboratory in Freiburg, proved contraction and relaxation of the stellate cells in vitro [35].

Golgi method and SEM after NaOH maceration reveal that the subendothelial processes of the stellate cells terminate as very thin spine-like microprojections. The striking observation is that these spines extend from the lateral edges of the subendothelial processes and direct obliquely through the space of Disse away from the abluminal surface of the endothelial cells to make contacts with the plasma membrane of the parenchymal cells. Thus, the hepatic stellate cells adhere to the endothelial cells and also to the parenchymal cells, as suggested by Kupffer.

(4) 'Relations of stellate cells to reticular fibers' (Kupffer; 1876, 1899) [4,5]

The hepatic stellate cells and reticular fibers are frequently stained concomitantly in gold preparations. Kupffer (1876) emphasized that stellate cells were associated with the reticular fibers which run along the sinusoid, but were not located within the thick bundles of connective tissue fibers. I supported his findings. The stellate cells surrounded with thick bundles of collagen fibers have no vitamin A-lipid droplets. Haruki Senoo and his group reported that the hepatic stellate cells were a primary site of collagen synthesis in the liver [36]. Mounting evidences demonstrated that activated stellate cells produce much extracellular matrix, losing stored vitamin A (Friedman, 1993) [37].

(5) 'Stellate cells in the stomach and intestine of a cat' (Rothe, 1882) [6]

It should be noted that Rothe found the stellate cells also in the stomach and the intestine of a cat. These findings indicate that the stellate cells distribute not only in the liver, but also in extrahepatic organs.

Yamada and Hirosawa in 1976 proposed a possible existence of vitamin A-storing system in the body [38]. We now know that the stellate cells, or vitamin A-storing cells, are distributed not only in the liver but also in other organs, such as pancreas, intestine, lung, heart, uterus, lymph node, etc. Thus it is interesting to note that Rothe already described stellate cells in extrahepatic organs.

I examined the vitamin A-storing system of the lamprey, *Lampetra japonica*, and found that vitamin A is stored specifically only in the mesenchymal cells of splanchynic organs, while it is scarcely stored in those cells of parietal organs (Wake, 1980) [22]. Based on these data, it is suggested that there exist two different categories of mesenchym in vertebrates; somatic mesenchym and splanchynic mesenchym. The mesenchymal cells of the former are conventional fibroblasts and those of the latter are stellate cells or vitamin A-storing cells. The stellate cell activation-associated protein (STAP) is expressed only in the splanchynic meseoderm (Nakatani et al. 2002) [39].

(6) 'Uptake of India ink or erythrocytes by capillary endothelial cells in the liver' (Kupffer, 1899) [5]

The incorportation of India ink by the sinusoidal endothelial cells was first explained by Kupffer, though he misunderstood the cell type of the sinusoid. Based on our careful examination of his figures, we supposed that his 'so-called endothelial cells' included Kupffer cells (liver macrophages). Controversy concerning the identity of different types of sinusoidal lining cells has been resolved by modern methods. Morphology, scavenging activity, origin and kinetics, and metabolic responses of Kupffer cells in normal and pathological conditions have been extensively investigated (Wake et al. 1989) [40].

On the other hand, since van Furth advocated that RES should be replaced by MPS or 'macrophage system', we have been trying to resolve a 'reticulo-endothelial confusion'. Is there no scavenger system other than macrophages in the body?

We have reexamined the lithium carmine vital staining of Kiyono, which contributed much to the development of the concept of the reticulo-endothelial system. Immunohistochemical and electron microscopic studies revealed that the dye was actively incorporated by sinusoidal endothelial cells in the liver (Kawai et al. 1998) [41] and reticular cells in the sinus of lymph node (Wake et al. 2001) [42]. Of note, uptake of the dye was comparatively much lower in macrophages and monocytes.

B–rd Smedsr–d (1985) in Troms–, Norway drew our attention to the specific elimination of an array of circulating soluble macromolecular waste products from the circulation by the sinusoidal endothelial cells [43].

On the third day of this Symposium, he will give us a Tutorial Lecture on the scavenger endothelial cells. It is now clear that there exist scavenger endothelial cells and scavenger reticular cells in blood and lymphatic circulations respectively.

## Conclusion

In conclusion, I can say clearly that the most striking contribution by Karl von Kupffer was his clear appreciation that the stellate cells existed in the liver tissue. He suggested that the stellate cells might be connective tissue cells in the hepatic lobule. His analysis has been linked to the fact that the hepatic stellate cells have a pivotal role in liver fibrosis and cirrhosis. The description of 'inclusion bodies' in the stellate cells has been playing an important in today's progress of theories of the vitamin A metabolism. Kupffer was also a pioneer of the research on the prominent scavenger activities of the sinusoidal wall. In addition, pupil Rothe contributed to the development of the modern concept on the vitamin A-storing cell system in vertebrate bodies.

Now I would like to finish my presentation by referring to Kupffer as an earnest teacher and academic.

Kupffer was good at making his lectures on difficult themes easy to understand, so naturally, he could attract the interest of his students.

His detailed anatomical scheme put up on the walls of lecture hall clarified the gist of his lectures, which impressed the students greatly.

As a person of delicate sensibility, Kupffer was refined in his effective speech at academic meetings. All of his lectures had power and remained deep in the memory of his audience. He always evaluated achievements of others very carefully. He was a critical reader of papers, however, his counter-argument never turned out to be a deep-rooted one. Personally, he seemed to be somewhat reserved, but once he opened his heart, he was very kind to every one.

Oscar Hertwig asked Kupffer to write a chapter on "Development of Central Nervous System" in his handbook. Kupffer accepted the offer, knowing that it would be his last work. He resigned his post as professor of Munich in October, 1901, and devoted himself to the writing. In September, 1902, when Kupffer almost completed the work, he had a stroke. For about three months, he sometimes got better but then had a relapse, and finally on the 16^th ^of December in 1902, his life ended at the age of 73, due to pneumonia which accompanied the stroke.

A century has passed since then, but his greatly inspiring achievements continue shining on in hepatology still today and, I'm sure, will remain in the memory of the people as ever.
